# VARIABLE PREDICTORS OF COGNITIVE PERFORMANCE WITHIN ETHNIC/RACIAL GROUPS USING A COMPUTERIZED COGNITIVE BATTERY

**DOI:** 10.1093/geroni/igac059.217

**Published:** 2022-12-20

**Authors:** Kacie Deters

**Affiliations:** University of California, Los Angeles, Inglewood, California, United States

## Abstract

The Computerized Cognitive Composite (C3) may provide an efficient assessment of early cognitive impairment to inform early interventions and screening criteria for Alzheimer’s disease clinical trials. The C3 battery, which includes the CogState Brief Battery, has components assessing memory, reaction time and aspects of executive function. The battery has little demand for spoken language and may reduce the sociocultural biases of traditional paper-and-pencil tests. The goal of this project was to determine demographic and genetic predictors of cognitive performance on the C3 battery across different ethnic/racial groups. We examined 4,026 cognitively normal participants (self-identified non-Hispanic Black, Asian, or White; Hispanic) at baseline visit from the Anti-Amyloid Treatment in Asymptomatic Alzheimer's Disease (A4) clinical trial. Linear models were performed to examine the association of C3 and years of age, years of education, gender/sex, and Apolipoprotein E (APOE) genotype, within ethnic/racial group. We found variation in demographic and genetic risk factors that predicted cognitive performance within ethnic/racial groups. These findings highlight the importance of within group analysis to identify risk factors for cognitive impairment.

